# Evaluation of the Potential Impact of a Multiplex Rapid Diagnostic Panel in Critically Ill Patients With Hospital-Acquired Pneumonia

**DOI:** 10.7759/cureus.21716

**Published:** 2022-01-29

**Authors:** Bradley J Erich, Abdullah Kilic, Elizabeth Palavecino, John Williamson, James Johnson, Christopher Ohl, Vera Luther, James Beardsley

**Affiliations:** 1 Department of Inpatient Pharmacy, The University of Kansas Health System, Kansas City, USA; 2 Department of Pathology, Atrium Health Wake Forest Baptist, Winston-Salem, USA; 3 Department of Pharmacy, Atrium Health Wake Forest Baptist, Winston-Salem, USA; 4 Department of Internal Medicine, Section of Infectious Diseases, Wake Forest School of Medicine, Winston-Salem, USA

**Keywords:** diagnostic stewardship, pcr, filmarray pneumonia panel, rapid diagnostic tests, hospital-acquired pneumonia

## Abstract

Background

Rapid diagnostic tools have emerged as valuable assets assisting clinicians in decision-making regarding patient management in the hospital setting. Our study sought to identify the potential impact of the BioFire® FilmArray® Pneumonia Panel (FP Panel) (BioFire Diagnostics, Salt Lake City, UT, USA) in patients with hospital-acquired pneumonia (HAP).

Methods

Respiratory samples obtained by bronchoalveolar lavage (BAL) or tracheal aspiration (TA) from ICU patients with a diagnosis of HAP were tested by the FP panel in addition to routine bacterial cultures. In addition, the electronic health records of these patients were reviewed to determine what potential changes in antimicrobial therapy could have been implemented if the panel results were known to the treatment team in real-time. A cost analysis was also performed incorporating the cost of the pneumonia panel and the savings associated with the potential decrease of antibiotic use and avoidance of the rapid viral diagnostic panel.

Results

Fifty-six patients met the study criteria. The FP panel results could have prompted a change in therapy in 36 (64.3%) patients, with an anticipated mean reduction in time to optimized therapy of approximately 51 hours. In addition, the panel identified three cases where antimicrobials should have been altered because patients were not receiving empiric therapy with activity against the causative pathogen and 34 opportunities for antibiotic de-escalation. The cost analysis calculated an additional cost of $10 per patient associated with using the FP panel.

Conclusions

The FP panel could have prompted a change in therapy in about two-thirds of patients studied. Its potential benefits include a more rapid time to optimized therapy, reduced exposure to and cost of broad-spectrum antimicrobials, and reduced cost of other rapid diagnostic tests.

## Introduction

This article was previously presented as a poster at ID Week 2021 (Virtual Event) in September 2021.

Hospital-acquired pneumonia (HAP) can be a severe and sometimes fatal infection. It accounts for up to 25% of all ICU infections. The overall mortality attributed to HAP can be as high as 30-50% [1]. Respiratory cultures are the current standard method of pathogen identification for HAP, but detection of pathogens and susceptibility reporting may take up to 72 hours. During this time, patients receive empiric broad-spectrum antimicrobial therapy that is typically tailored once culture results are available. The timeliness of these critical therapy changes is dependent on the rapidity of microbiology results. Recently published guidelines suggest that the use of initial multiplex testing for HAP may help reduce the use of unneeded antibiotics and other diagnostic tests [2].

The BioFire® FilmArray® Pneumonia Panel (FP Panel) (BioFire Diagnostics, Salt Lake City, UT, USA) is a multiplex polymerase chain reaction (PCR) panel that is capable of detecting 18 bacteria, eight viruses, and seven antimicrobial resistance genes within approximately one hour of testing [3]. This test is designed to identify pathogens from respiratory samples, including bronchoalveolar lavage (BAL), tracheal aspirate (TA), sputum, and mini-BAL samples, but costs more than traditional respiratory cultures. However, previously published literature has shown that the FP panel compares well to respiratory cultures when detecting bacteria, viruses, and antimicrobial resistance genes [4-8].

A previous study that retrospectively evaluated patients with HAP found the potential for antibiotic adjustment in 70.7% of patients based on results of the FP panel; however, no analysis of the test’s potential cost impact was provided [9]. Therefore, prior to implementing this new test at our institution, we sought to evaluate its possible impact by assessing the potential clinical and cost implications had the panel results been known to the treatment team in real-time.

## Materials and methods

This was a retrospective, single-centered, IRB-approved study performed at a large academic medical center in the Southeast United States. The study’s general schema was to identify ICU patients who had respiratory samples collected for routine culturing, to test these samples using the FP panel, and then to retrospectively determine the potential impact that the FP panel could have had if the results were available to the treatment team during the care of the patient. Specific pathogens that the FP panel is capable of detecting are listed in Table 1. To complete this assessment, the microbiology laboratory information system (LIS) was queried to identify patients with the following characteristics: (1) location at the time of culture of the medical ICU (MICU), surgical ICU (SICU), or trauma ICU (TICU); (2) collection of a TA or BAL sample obtained from November 1, 2019, through February 29, 2020; and (3) age greater than or equal to 18 years.

Patients who did not meet the clinical criteria for HAP as defined by the Infectious Diseases Society of America [10] were excluded. Based on the estimated relative frequency of patients diagnosed with HAP at our institution, we targeted evaluating positive respiratory cultures with the following geographic distribution: 20 samples from the MICU, 15 samples from the SICU, and 10 samples from the TICU. We additionally targeted five negative respiratory cultures from each of the above clinical units.

**Table 1:** Patient characteristics. TA: Tracheal aspirate; BAL: Bronchoalveolar lavage; RVP: Respiratory virus panel. ^1 ^Positive RVP results included adenovirus, rhino/enterovirus, and respiratory syncytial virus. The FilmArray Pneumonia Panel® detected these viruses in all four cases.

Characteristic	Results (N = 56)
Age, years, mean (± SD)	60.4 (± 15.9)
Males, n (%)	39 (70)
Type of culture, n (%)	
TA	46 (82.1)
BAL	10 (17.9)
RVP, n (%)	
Obtained	36 (64)
Positive^1^	4 (7)

The manufacturer provided a limited number of FP panel tests for this evaluation. Respiratory samples from each ICU were evaluated in the chronological order that they were collected until the target sample size for each category was reached. The samples of patients meeting inclusion criteria were selected for extra testing with the FP panel in addition to routine bacterial cultures. Samples were saved (frozen) under guidance from BioFire regarding storage timing and environment and in accordance with institution-specific microbiology laboratory storage requirements for respiratory specimens. The electronic health records (EHR) of these patients were reviewed retrospectively. Patients who did not have HAP were excluded, and another patient from the same category was evaluated. No patient was included in the study more than once. The primary outcomes of the study were the number of instances where the FP panel results could have prompted a change in antimicrobial therapy and the potential reduction in time to optimized therapy for these patients if the treatment team had acted on the results in real-time. The secondary outcomes were the correlation of the panel test results with respiratory cultures, the identification of units where the results would have had the highest impact, and the cost impact of using the FP panel.

Two investigators performed an EHR review of included patients to determine the potential changes in antimicrobial therapy if the results of the panel had been known in real-time. If the two investigators disagreed on their assessment, the case was adjudicated by a third study team member. Our analysis included the supposition that the panel test results would be available and actionable at the time of the respiratory sample collection, plus four hours allowed for test processing. When assessing the potential changes in antimicrobial therapy, our study team assumed the “best-case scenario” for antimicrobial decision-making. By “best-case scenario,” we assumed that after the FP results were available, the provider would have adjusted antimicrobial coverage to provide therapy against any untreated pathogens and to discontinue anti-methicillin-resistant Staphylococcus aureus (MRSA) therapy if MRSA was not detected. We did not assume immediate discontinuation of Gram-negative antimicrobial therapy on the basis that the panel did not include all the pathogenic Gram-negative organisms implicated in HAP (e.g., Morganella morganii, Burkholderia cepacia). We defined the time difference to optimized therapy as the time of the final culture susceptibility report minus the time of the availability of the FP panel report for those cases where the FP panel results would have prompted a change in therapy. 

A cost analysis was performed to determine the potential impact of implementing the panel by subtracting the wholesale-acquisition cost (WAC) of antimicrobials that could have been avoided due to discontinuation/de-escalation and the cost of avoided respiratory viral panel (RVP) tests from the cost of the FP panel test.

## Results

Eighty-six patients were identified from the microbiology LIS; 30 were subsequently excluded due to not meeting the clinical criteria for HAP, leaving 56 patients who met study criteria. Patient characteristics are provided in Table 2, and their location distribution is listed in Table 3. The target sample allocation was not achieved because of a limited number of test kits and a higher than anticipated number of exclusions.

**Table 2:** Organisms and resistance genes detectable by the FP panel. FP panel: FilmArray® Pneumonia panel.

Bacteria (Semi-quantitative)	Antibiotic Resistance Genes
Acinetobacter baumannii-calcoaceticus complex, Enterobacter cloacae, Escherichia coli, Haemophilus influenzae, Klebsiella aerogenes, Klebsiella oxytoca, Klebsiella pneumoniae group, Moraxella catarrhalis, Proteus spp., Pseudomonas aeruginosa, Serratia marcescens, Staphylococcus aureus, Streptococcus agalactiae, Streptococcus pneumoniae, Streptococcus pyogenes	ESBL CTX-M
	Carbapenemases: KPC, NDM, OXA-48-like, VIM, IMP
	Methicillin-resistance: mecA/mecC and MREJ
Atypical bacteria	Viruses
Legionella pneumophila, Mycoplasma pneumoniae, Chlamydia pneumoniae	Influenza A and B, Adenovirus, Coronavirus, Parainfluenza virus, Respiratory Syncytial virus, Human Rhinovirus/Enterovirus, Human Metapneumovirus, Middle East Respiratory Syndrome Coronavirus (MERS-CoV)

**Table 3:** Patient location of respiratory samples used in analysis. ^a ^Did not meet target sample allocation. MICU: Medical intensive care unit; SICU: Surgical intensive care unit; TICU: Trauma intensive care unit.

Location	Positive Culture	Negative Culture	Total
MICU	20	4^a^	24
SICU	15	5	20
TICU	7^a^	5	12

The FP panel would have prompted a change in antimicrobial therapy in 36 cases (64.3%). These instances included three cases where the FP panel indicated the need to expand antimicrobial coverage because the patient’s empiric therapy did not have the activity to treat the pathogen identified by the FP panel. These pathogens were influenza A, Extended-spectrum beta-lactamase (ESBL)-producing E. coli, and Enterobacter cloacae complex. In addition, there were opportunities to narrow antibiotics in 34 cases (60.7%) based on FP panel results (one case had opportunities to both expand and narrow antibiotics). The most common antibiotic narrowing change would have been to discontinue empiric vancomycin. For the 36 cases potentially impacted by the FP panel, the mean (±SD) difference in time to optimized therapy would have been 51 ± 30.4 hours.

The organisms identified by the FP panel and respiratory culture were in exact agreement in 25 cases (44.6%). The FP panel identified additional organisms in 27 of the other 31 cases (48.2% of the 56 samples tested). In seven cases (12.5%), the respiratory culture identified bacteria not detected by the FP panel; all of these organisms were not included in the FP panel. In five of these cases, the bacteria (Achromobacter xylosoxidans, Stenotrophomonas maltophilia, and M. morganii) were thought to be pathogenic. Based on analysis of patient location, the panel would have been impactful for patients in all ICUs, with the most significant effects in patients in the TICU (Table 4).

**Table 4:** Cases where the FP panel would have prompted a change in therapy. MICU: Medical intensive care unit; SICU: Surgical intensive care unit; TICU: Trauma intensive care unit; FP panel: FilmArray® Pneumonia panel.

Location	Cases With Potential Therapy Change, n (%)
MICU	13/24 (54.2)
Positive culture	11/20 (55)
Negative culture	2/4 (50)
SICU	12/20 (60)
Positive culture	9/15 (60)
Negative culture	3/5 (60)
TICU	11/12 (91.7)
Positive culture	6/7 (85.7)
Negative culture	5/5 (100)
All Patients	36/56 (64)
Positive culture	26/42 (62)
Negative culture	10/14 (71)

The cost of each FP panel was anticipated to be $195 (USD), and the potential cost avoidance normalized per patient from discontinuing RVPs would have been $125. The mean antibiotic savings per patient was calculated to be $60, resulting in an estimated additional cost of $10 per patient using the FP panel.

## Discussion

We found that approximately two-thirds of patients could have had their antimicrobials optimized if the results of the FP panel were known in real-time. While most of these changes were for antimicrobial de-escalation, the panel also identified cases where patients were not receiving active therapy against the detected pathogens, and antibiotic escalation would have been required. Access to timely antimicrobial information on which to make these changes in therapy can enhance the care of patients with HAP. Studies have shown that antimicrobial therapy is not active against causative pathogens, leading to worse patient outcomes [11-16]. Being able to detect the need for expanding therapy to target untreated pathogens within a few hours can lead to more rapid administration of adequate therapy.

Current guidelines recommend that empiric antimicrobial therapy be de-escalated based on the results of microbiologic studies performed on respiratory samples [1]. The availability of microbiologic data from the FP panel would allow antibiotic de-escalation to occur much sooner than when relying solely on standard respiratory cultures. This would lead to reduced antibiotic expenditures and adverse effects, and it may also lead to reduced antimicrobial resistance. The most frequent potential de-escalation prompted by the FP panel in our study was the discontinuation of vancomycin. Early discontinuation of vancomycin could be associated with a reduction in the development of acute kidney injury (AKI), as many studies assessing patients receiving concomitant vancomycin and beta-lactam antibiotics have shown that AKI may develop within two-to-three days of therapy [17-19]. With this in mind, it can be helpful to leverage and act upon available rapid diagnostic technology such as the FP panel to optimize antimicrobial therapy and avoid potential adverse events.

Our results are consistent with those of Buchan BW et al. [9]. In a study of 259 patients with respiratory samples obtained by either BAL or mini-BAL, these investigators concluded the FP panel had the potential to modify antimicrobial therapy in 70.7% of patients if the panel results were available to the treatment team. Similar to our investigation, the most common potential antibiotic modification in their study was antimicrobial de-escalation/discontinuation.

Appropriately incorporating the FP panel into clinical practice requires an understanding of potential discrepancies between the FP panel and standard respiratory culture results. For example, because the FP panel includes targets that identify only a limited number of organisms, there will be cases where organisms that were not detected by the panel will grow out on culture. Likewise, because the FP panel can detect organisms at a relatively low concentration, including those organisms that are difficult to grow or are no longer alive, it is not unexpected that the panel will detect some organisms that do not grow out on culture. Similar to our study, this phenomenon has occurred frequently in other investigations [4,6,7,9].

Although there is an increased cost associated with using the FP panel, this cost increase is minimal when considering the cost savings associated with avoiding antimicrobials and RVP tests. Of note, in our study, we anticipated that concomitantly ordered RVPs would not be needed if the patient was tested with the FP panel since the FP panel, like the RVP, contains the most important respiratory viruses, excluding SARS-CoV-2. However, since this study occurred prior to the COVID-19 pandemic, it is impossible to determine how the need to test for SARS-CoV-2 would have impacted our analysis. Also, this study was conducted at an institution that does not routinely order MRSA nasal swabs to aid in the de-escalation of anti-MRSA antibiotics. Therefore, our results may not reflect the potential impact of the FP panel at an institution where MRSA nasal swabs are common practice.

This study had limitations worth mentioning. It was a single-center study, and the evaluation of HAP was based on retrospective evaluation of clinical documentation. A limited number of tests were available for evaluation, which prevented attaining the targeted sample size. As with all tests, it is not the availability of test results but the response to those results which affects patient care. We assumed a best-case scenario where an appropriate therapeutic decision would be made four hours after respiratory sample collection. We cannot guarantee that such action would actually occur after test implementation. However, procedures can be implemented which promote a prompt response to results from rapid diagnostic tests.

Because we wanted to compare the pathogen identification characteristics of the FP panel and standard cultures, we designed our study to focus primarily on positive respiratory cultures. As a result, our study included a higher percentage (75%) of positive cultures than typically found in clinical practice. However, since the potential for therapy change was fairly similar for positive and negative cultures (62% vs. 71%, respectively), we would anticipate that altering the ratio of positive to negative cultures would have only a minor impact on our results. In addition, the cost analysis did not include expenses related to ICU or hospital length of stay or treatment of adverse events. Also, the study used WAC, not actual purchasing price, for the financial analysis.

Lastly, since our study only evaluated patients with documented HAP, it is possible that the full potential for changes in therapy was underestimated. For example, empiric antibiotics are often ordered for suspected lung infections that do not meet a strict definition of HAP. It is possible that the FP panel may prove to be beneficial when applied to these clinical scenarios as well.

## Conclusions

The FP panel could have improved time to optimal antimicrobial therapy in approximately two-thirds of the patients with HAP in our study by an average of approximately two days. This new rapid diagnostic test may significantly impact the care provided to patients with HAP in the ICU by decreasing the time to optimized therapy and supporting antimicrobial stewardship efforts. The total cost impact of implementing this test is anticipated to be minimal. Based on the results of this analysis, our institution has decided to proceed with planning to implement the FP panel into the care of our critically ill adult patients.
